# VEGF, a Key Factor for Blood Brain Barrier Injury After Cerebral Ischemic Stroke

**DOI:** 10.14336/AD.2021.1121

**Published:** 2022-06-01

**Authors:** Yue Hu, Yangmin Zheng, Tao Wang, Liqun Jiao, Yumin Luo

**Affiliations:** ^1^Institute of Cerebrovascular Disease Research and Department of Neurology, Xuanwu Hospital of Capital Medical University, Beijing, China.; ^2^Department of Neurosurgery, Xuanwu Hospital of Capital Medical University, Beijing, China.; ^3^Beijing Geriatric Medical Research Center and Beijing Key Laboratory of Translational Medicine for Cerebrovascular Diseases, Beijing, China.; ^4^Beijing Institute for Brain Disorders, Capital Medical University, Beijing, China.

**Keywords:** BBB, cerebral ischemia, VEGF, brain

## Abstract

Blood brain barrier (BBB) injury is an important factor affecting the prognosis of ischemic stroke. Extensive research on BBB injury has revealed that blood vessels and neural networks are interdependent and interrelated during and after the development of the brain. An array of signaling molecules, known as angioneurins, can affect both blood vessels and neural networks simultaneously. Angioneurins not only regulate the angiogenesis and remodeling process of the vascular system, but also act as neurotrophic and neuroprotective factors, or serve as guide molecules for axons. Vascular endothelial growth factor (VEGF) is a type of angioneurin that is expressed in neurons, astrocytes, macrophages, and vascular endothelial cells in ischemic and hypoxic brain tissues after cerebral ischemia. VEGF can increase and induce the destruction of the endothelial barrier in the early stages of cerebral ischemia. Both the upregulation of endogenous VEGF levels and the use of exogenous VEGF are harmful in the acute stage of stroke. However, the harmful effects of VEGF on vascular integrity are transient. Several studies have shown that VEGF regulates angiogenesis, neurogenesis, neurite growth and brain edema after cerebral ischemia. Therefore, it is crucial to understand the dual role of VEGF in ischemic stroke. The following will focus on the damage caused by VEGF to the BBB in the context of cerebral ischemic stroke, as well as therapeutic studies targeting VEGF.

The blood brain barrier (BBB) is a complex structure located between cerebrovascular and brain tissues. Structurally, it is characterized by a physical barrier formed by endothelial tight junctions and a transport barrier formed by membrane transporters and vesicular mechanisms. The BBB is a dynamic component of the brain-vascular interface that maintains brain homeostasis and regulates solute penetration into the brain tissue [1].With continuous progress of research on BBB injury, it has been found that blood vessels and neuronal networks are closely related and depend on each other during the development and after the maturation of the brain. After cerebral ischemic hypoxia occurs, the obvious influence of typical angioneurins, such as VEGF, angiopoietins (Ang1, Ang2), angiopoietin-like proteins (Angptl4) and erythropoietin, on the BBB can be noted. Different types of angioneurins have different effects on the BBB. Ang1, Angptl4 and erythropoietin can protect the BBB after cerebral ischemia, whereas Ang2 and VEGF damage the BBB. It has been speculated that the maintenance of BBB requires a balance among VEGF, Ang 1 and Ang 2, and cerebral ischemia may lead to the disruption of this balance, leading to BBB injury [2]. VEGF, a pivotal type of angioneurin plays an important role in the occurrence, development and prognosis of cerebral ischemia.

Upregulation of endogenous VEGF levels and the use of exogenous VEGF are harmful after acute stroke. This may be related to VEGF-mediated BBB rupture and vascular leakage, resulting in brain edema, increased intracranial pressure and neuroinflammation. However, the deleterious effects of VEGF on vascular integrity are transient, and both VEGF preconditioning [3] and VEGF treatment after the acute phase [4, 5] have neuroprotective effects. Therefore, the time point at which VEGF takes effect is the key to understanding its role in ischemic stroke. In addition, a single lateral intraventricular injection of VEGF protects against cerebral ischemia without affecting BBB permeability [6].The mode and location of VEGF administration are other key factors that influence its effect on ischemic encephalopathy. Considering that VEGF damages the BBB in the acute stage of cerebral ischemia and protects the brain tissue in the recovery stage of cerebral ischemia, the time, dose and route of administration of VEGF after cerebral ischemia should be considered comprehensively to ensure that VEGF plays a protective role and to avoid adverse effects. Some studies have found that the upregulation of endogenous VEGF expression and systemic administration of VEGF-A to the level of angiogenesis induce BBB leakage to produce harmful effects after 24 h of ischemic stroke. However, low doses that do not produce angiogenesis (e.g., via cerebral arteries) or lateral ventricles or local administration may confer neuroprotective effects, even during the acute phase of stroke. Increased VEGF-A dose is associated with neuroprotective effects even in the acute phase that occurs more than 24 h after stroke [7].

VEGF receptor (VEGF-R) 2 (VEGF-R2) is mainly expressed in neurons and vascular endothelial cells, whereas VEGF-R1 is mainly expressed in the vasculature such as choroid and glial cells. Hence, VEGF acts on not only vascular endothelial cells but also other cells. VEGF is mainly involved in angiogenesis and acts directly on neurons, leading to neurite extension, neuroprotection, and neurogenesis [8]. Therefore, it is necessary to explore a therapeutic strategy that can not only avoid damage caused by VEGF to the BBB but also exert its protective effect on the blood vessels and nerves of the brain tissue. To this end, based on the existing literature, we aimed to discuss the current knowledge on the damage caused by VEGF to the BBB in the context of cerebral ischemia as well as therapeutic studies targeting VEGF.

## Temporal and spatial expression of VEGF in animal models of cerebral ischemia

VEGF mRNA began to be upregulated in the peripheral ischemic area at 3 h after middle cerebral artery occlusion (MCAO) in rats, reached a peak at 24 h and remained at a lower level until 7 days after MCAO. Double labelling experiments have shown that microglia/macrophages are the main cell types that express VEGF [9]. Meanwhile, another study found that the expression of VEGF receptor flt is induced in endothelial cells during angiogenesis in infarcted brain tissue in rats from 3 h to 3 weeks after MCAO [10]. However, the expressions of VEGF mRNA and protein were rapidly induced after transient MCAO (tMCAO) in rats. Neurons and pial cells are the main sources of VEGF expression. VEGF mRNA and protein were clearly expressed at 1 h and peaked at 3 h after tMCAO in rats but continued to be expressed in pial cells in the middle cerebral artery region for 3-7 days [11]. Another study found significantly different expression levels of VEGF in the models of chronic cerebral ischemia. Semi-quantitative reverse transcriptase-polymerase chain reaction analysis showed that the quantity of VEGF mRNA in the brain tissue showing chronic cerebral hypoperfusion was significantly increased at 24 h, peaked at 7 days, decreased at 21 days and returned to the baseline level at 90 days after operation in rats. Western blot analysis showed that the expression of VEGF protein in rats with brain tissue showing chronic cerebral hypoperfusion (model group) increased from 24 h to 21 days after operation and decreased to the level of the control group after 90 days. Immunohistochemical analysis showed that VEGF was mainly expressed in the endothelial cells. Sustained VEGFR-1 expression was upregulated, whereas VEGFR-2 expression was not [12]. In conclusion, the temporal and spatial expressions of VEGF are completely different in models of acute and chronic cerebral ischemia. One study specifically compared transient and permanent MCAO (pMCAO) in rats and showed that VEGF-A (expressed in neurons and endothelial cells), VEGFR-1 (expressed in neurons, endothelial cells and astrocytes) and VEGFR-2 (expressed in endothelial cells and astrocytes) were all elevated during 1 to 3 days. These changes were more pronounced in the diseased hemisphere and after pMCAO [13]. In addition to tMCAO and pMCAO inducing different ways of VEGF expression, global and focal transient cerebral ischemia also induce different ways of VEGF expression, as shown in Table 1.

**Table 1:** Expression of VEGF at different time and location in rat models of transient focal and global cerebral ischemia.

Spatiotemporal expression of VEGF	Transient focal cerebral ischemia [10, 11, 13]	Transient global cerebral ischemia [14]
Initiation time	1 h after reperfusion	12 h after reperfusion
Peak time	3 h to 1 day after reperfusion	1 to 2 days after reperfusion
Location	Cortical neurons, endothelial cells and pial cells at the infarct area and glial cells at the edge of the infarct area	Astrocytes and neurons in the hippocampus

## The mechanism of VEGF -mediated BBB disruption

The effect of VEGF on BBB injury after cerebral ischemia is not completely clear. Both in vitro and in vivo studies have shown that increased activity of matrix metallopeptidase 9 (MMP-9) appears to be a key molecular mechanism for VEGF -mediated BBB disruption after ischemia [15-17]. There was a significant positive correlation between VEGF and MMP-9 levels in the plasma of stroke patients during acute stroke [16]. And VEGF can increase the activity of MMP-9. MMP-9 regulates hypoxia-induced vascular leakage in the brain tissue by degrading the neurovascular matrix and rearranging tight junction proteins, leading to BBB rupture, brain edema and hemorrhage [18]. Early intracerebral injection of anti-VEGF neutralizing antibody can significantly reduce the expression of VEGF in tMCAO rats, improve neurological prognosis, and reduce infarct size and BBB leakage by reducing MMP-9 expression [19].The decrease in VEGF-A and MMP-9 derived from astrocytes is related to an increase in tight junction protein expression. Therefore, reducing the levels of VEGF-A and MMP-9 relieves BBB damage and improves the neurological deficit score of ischemic brain tissue in MCAO rats [20].

Some studies have focused on the effect of VEGF on the BBB structure. VEGF induces BBB hype-rpermeability by disrupting tight junction regulatory proteins including occludin (OCLN) and claudin (CLN) [21]. An in vitro study on bovine brain microvascular endothelial cells showed that VEGF increased paracellular permeability and decreased transendothelial electrical resistance (TEER), which was related to the loss of zonula-occludens (ZO)-1(ZO-1) and OCLN [22]. In vitro BBB models of co-culture of cerebral microvascular cells, astrocytes and neurons demonstrated that ischemic neurons induce astrocyte-derived VEGF production, resulting in endothelial barrier injury accompanied by loss of tight junction proteins OCLN and CLN-5 [23]. Other studies have identified a new signaling pathway to explain how VEGF induces vascular leakage. VEGF activation promotes rapid endocytosis of beta-arrestin2-dependent VE cadherin (an important endothelial cell adhesion molecule) and internalization of VE cadherin into clathrin-coated vesicles, followed by breakdown of intercellular junctions, resulting in the destruction of the endothelial barrier [24, 25]. Endothelial transmembrane tight junction proteins CLN-5 and OCLN are the main targets of VEGF-A. With regard to establishing an animal model of experimental autoimmune encephalomyelitis, it was found that the downregulation of CLN-5 and OCLN was accompanied by the upregulation of VEGF-A and was associated with BBB injury [26]. VEGF and its receptor VEGFR-2 are both important BBB regulators of permeability. It has been found that 10% hypertonic saline may reduce ischemia-induced BBB disruption by inhibiting the downregulation of VEGF-VEGFR2-mediated expressions of ZO-1 and CLN-5 in vitro studies [27].

## VEGF participates in the destruction of BBB after rtPA thrombolytic therapy

Thrombolytic therapy with tissue plasminogen activator (tPA) increases BBB permeability and the risk of hemorrhage transformation. Delayed tPA treatment can promote the expression of VEGF and activation of MMP-9 in the BBB [28]. VEGF mediates endothelial endocytosis and leads to a temporary increase in BBB permeability after ischemic stroke in mice, which results in BBB damage and hemorrhage transformation [29]. Some studies have found that an intravenous injection of anti-VEGF neutralizing antibody/VEGF receptor antagonists can inhibit hemorrhage transformation [28]. In conclusion, inhibition of VEGF induction may play a beneficial role in thrombolytic therapy with recombinant tPA (rtPA) after stroke and by alleviating post-treatment hemorrhage [30].

In addition, some studies explored the interference of the VEGF signaling pathway with the BBB at the molecular level and found that lack of cathepsin K can aggravate rtPA -induced hemorrhage transformation after cerebral ischemia, which leads to neurological impairment and neuronal apoptosis. Further studies have shown that cathepsin K can improve the safety of rtPA in the treatment of stroke by regulating the VEGF signaling pathway [31]. Another study found that recombinant ADAMTS13 (a disintegrin and metalloproteinase with a thrombospondin type 1 motif, member 13) inhibited the up-regulation of VEGF in vascular endothelial cells after stroke through the Akt/rhoa-mediated VEGF pathway, regulated BBB integrity and reduced tPA -induced hemorrhage transformation [32].

## VEGF aggravates BBB injury when stroke is associated with other comorbidities

Diabetes as a comorbidity in patients with stroke aggravates BBB injury, and brain edema is the main pathological manifestation of diabetic stroke. Studies have found that a potential mechanism by which diabetes increases BBB injury is the activation of VEGF signaling. Expression levels of VEGF-A and VEGF-R2 were found to be significantly higher in ischemic brain tissue in the diabetic stroke rat model than in non-diabetic stroke model, and the mRNA level of VEGF-R2 was positively correlated with the level of brain edema, but not with the volume of cerebral infarction, in the former model [33]. Reeson et al. established a T1MD stroke rat model and found that the expression of VEGF-R2 was continuously increased in the vascular network around the infarcted brain tissue, and that abnormal VEGF signaling resulted in BBB injury, thereby limiting the functional recovery after stroke [34]. Overexpression of VEGF in diabetic stroke models can potentially induce massive vascular injury [35, 36], and inhibition of VEGF can reinforce tight junction proteins and reduce endothelial leakage, that is, BBB leakage. Therefore, inhibition of VEGF gene expression or inhibition of VEGF signaling with drugs can reduce vascular dysfunction and improve stroke recovery in diabetic animals [37]. Treatment of T1DM stroke rats with APE1/Ref-1 redox inhibitor APX330 reduced VEGF expression in the margin of the ischemic brain, suppressed dysfunctional angiogenesis and relieved BBB leakage. Consequently, regulation of VEGF expression provides a new treatment strategy for diabetic stroke [38].

In addition, obesity as a comorbidity in patients with stroke also exacerbates BBB destruction. Il-doo et al. established the tMCAO models in obese and non-obese mice (obesity induced by a high-fat diet) and found that BBB damage was higher in obese mice than in non-obese mice. These results were related to the increased expression of VEGF-A and VEGF-R2, and inhibition of VEGF and VEGF receptor expression could reduce BBB damage in the context of stroke comorbid with obesity. Aflibercept decreased VEGF-A gene expression in obese mice, reduced brain edema and BBB disruption without changing infarct size and imparted no adverse effects on malignant infarcts in non-obese mice. It was found that VEGF signaling is overactivated in the context of stroke comorbid with obesity, and inhibition of VEGF signaling can alleviate BBB damage according to this study. Hence, selective regulation of VEGF expression can reduce vascular permeability and brain edema in obese mice, which also provides a new therapeutic direction for obese patients with acute stroke [39].

## Therapeutic studies targeting VEGF

The application of VEGF in the early stage of cerebral ischemia increases BBB permeability as well as hemorrhage transformation, and many studies have reduced BBB injury by inhibiting VEGF-mediated vascular leakage. Ang-1 regulates the expression of ZO-2 and counteracts VEGF-induced vascular permeability [40]. Moreover, studies have found that the combined use of Ang-1 and VEGF in the early stage of cerebral ischemia can promote mature neovascularization and protect damaged cells without affecting BBB permeability. In conclusion, early intraventricular injection of rAAV-VEGF and rAAV-Ang1 may be a good experimental gene therapy strategy for stroke [16, 41, 42]. Angptl4 can suppress the upregulation of VEGF in the vascular endothelium after stroke and relieve the increase in BBB permeability caused by cerebral ischemia and thrombolytic therapy. These results suggest that Angptl4 may be a promising target molecule for vascular protection after thrombolytic therapy [43, 44]. It should be noted that the combination of VEGF and Ang-2 promotes angiogenesis better than does VEGF alone; however, the combination of VEGF and Ang-2 may lead to BBB destruction [45]. Novel Src kinase inhibitors decrease BBB damage by inhibiting VEGF-mediated vascular leakage [46]. The use of selective integrin α -β3 inhibitors after stroke may reduce BBB rupture of focal ischemia by inhibiting VEGF-mediated vascular rupture [47-49]. Fc-saxatilin can reduce VEGF-induced permeability in human microvascular endothelial cells (HBMECs) by regulating the activation of Src and Fak signaling proteins in downstream signaling pathways, which may contribute to its protective effect on ischemic cerebrovascular leakage [50].

Some studies have suggested that the BBB can be protected by inhibiting VEGF or its receptor expression to reduce damage to the BBB structure. Anti-IL-9 neutralizing antibody can repair damaged tight junction proteins and induce decreased VEGF-A expression in ischemic brain tissue, thus reducing BBB damage by inhibiting VEGF-A-mediated endothelial barrier structure injury [51]. Juglanin decreased the mRNA and protein expression of VEGF and VEGF-R2 in MCAO mice and restored the normal expression of two important tight junction proteins OCLN and ZO-1, thereby decreasing BBB permeability and protecting tight junction function by inhibiting the VEGF/VEGF-R2 signaling pathway [52]. In the acute phase of stroke, recombinant erythropoietin reduces damage to the BBB structure by downregulating VEGF-R2 expression and the response to VEGF signaling. Therefore, erythropoietin can protect against BBB injury caused by focal cerebral ischemia in mice [53]. Src protein tyrosine kinase (PTK) inhibitors may protect the rat brain from ischemic injury by decreasing VEGF-A expression and upregulating CLN-5 expression, thereby preserving the integrity of BBB structure. These results suggest that inhibition of Src phosphorylation can reduce BBB damage after transient focal cerebral ischemia in rats [54].

Hypoxia-inducible factor (HIF) is a transcription factor that binds to the VEGF promoter to initiate and activate the transcription of VEGF genes directly [55]. Under hypoxic conditions, HIF activates the VEGF gene, which allows cells to adapt to hypoxic conditions [56].Thus, regulating HIF can affect VEGF expression and thereby regulating BBB permeability. Many studies have demonstrated that inhibition of HIF can suppress VEGF expression and thereby alleviating BBB injury. Ligustilide, the main active ingredient in Chuanxiong rhizome, can ameliorate the permeability of the BBB model in vitro as a result of induction by oxygen-glucose deprivation (OGD) through the HIF-1α/VEGF pathway [57]. Sirt3 protein controls VEGF expression by inhibiting HIF-1α signaling after OGD. Sirt3 knockout mice showed severe BBB damage and inflammatory responses in the acute phase of pMCAO. Sirt3 plays a protective role in ischemic stroke by regulating HIF-1α/VEGF signaling pathway [58]. Inhibition of HIF-1α with its inhibitor YC-1 can significantly decrease VEGF upregulation in neurons and reduce ischemia-induced BBB damage during acute cerebral ischemia [59]. β2 adrenergic receptor (β2-AR) antagonist alleviates ischemia-induced BBB injury by inhibiting HIF-1α expression and decreasing VEGF upregulation and the secretion of VEGF and MMP-2 [60]. However, Shi et al. found that HIF-1α can induce the production of stress-induced proteins Sestrin2 and VEGF in severe hypoxic cerebral ischemia, but Sestrin2 plays a leading role in protecting the BBB of neonatal rats with severe hypoxic ischemia by inhibiting VEGF signaling. However, HIF-1α only induces VEGF production under moderate hypoxia, and the use of HIF-1α agonists can increase BBB permeability through VEGF signaling. Overall, under different hypoxic conditions, HIF can protect or destroy the BBB by inhibiting or inducing VEGF production [61]. In conclusion, HIF expression should be regulated according to the degree of hypoxia, and VEGF expression should be adjusted to protect the BBB.

Ischemic stroke is a complex disease with multiple underlying mechanisms; thus, combination therapy provides a better combination of neural and vascular protection with the progression of treatment. While N-acetylcysteine (NAC) alone shows protective effects on reperfusion injury, a combination of NAC and normobaric hyperoxia (NBO) significantly inhibits the induction of VEGF in ischemic brain tissue and reduces tight junction protein degradation. The results of this study suggest that a combination of NAC and NBO therapy can effectively attenuate BBB injury and significantly improve the prognosis of brain injury after cerebral ischemia [62].

Because of the dual role of VEGF, the dose, timing, and route of administration of VEGF therapy are the key factors involved in determination of the risk of ischemic BBB damage and cerebral edema. Consequently, these three factors must be considered while applying VEGF in the treatment of ischemic stroke. While VEGF-B is less effective than VEGF in terms of angiogenesis, it still has neuroprotective and regenerative effects; the application of VEGF splice isoform VEGF165b, which has neuroprotective and anti-angiogenic properties, can partially avoid the dual effect of VEGF and allow more flexible drug administration and treatment [2].

## Perspectives

Studying the destructive effect of VEGF on the BBB after cerebral ischemia and designing the corresponding regulation methods are of great significance for the treatment of ischemic encephalopathy. Several studies have reported on the use of VEGF to prevent BBB injury after cerebral ischemia. Because tPA thrombolytic therapy after stroke can increase the permeability of the BBB as well as the risk of hemorrhage transformation, inhibition of VEGF induction has broad application prospects after delayed tPA treatment. However, the treatment of stroke based on VEGF is mostly studied in animal experiments, and no clinical evaluation of this treatment strategy is currently available. Owing to the dual effects of VEGF after cerebral ischemia, clinical evaluation will face many difficulties. When the angiogenic and neuroprotective properties of VEGF are needed, the vasogenic permeability and BBB damage caused by VEGF should be considered, and vice versa. Current clinical studies on VEGF, retinal neovascularization disease and glioblastoma multiforme are the only neurological diseases that have been clinically approved for VEGF- targeted therapy. Thus, clinical researchers must increase their efforts to accumulate a large amount of data on the destructive mechanisms associated with BBB permeability after cerebral ischemia, the administration mode and protective mechanisms of ischemic brain tissue against VEGF. Therefore, we will explore personalized administration schemes for different types of ischemia, which can not only exert the neuroprotective effect of VEGF but also overcome the shortcomings of BBB leakage and brain edema, furthermore, we will provide new therapeutic ideas and methods for the treatment of ischemic cerebrovascular disease.
